# Biodiversity and Winemaking Characteristics of Yeasts Isolated from *Docynia delavayi* (Franch.) Schneid. Wine Microbiota

**DOI:** 10.3390/foods14040553

**Published:** 2025-02-07

**Authors:** Ling Zhu, Zhangxing Li, Yupeng Liang, Xiu Gao, Qingfang Xu, Weiliang Liu, Lifang Zhang, Jian Cai

**Affiliations:** 1 Yunnan Engineering Research Center of Fruit Wine, Qujing Normal University, Qujing 655011, China; lingzhu2021@mail.qjnu.edu.cn (L.Z.); appleartgao@163.com (X.G.); 101022@mail.qjnu.edu.cn (Q.X.); liuweiliang@mail.qjnu.edu.cn (W.L.); lifangz6933@mail.qjnu.edu.cn (L.Z.); 2 Faculty of Food Science and Engineering, Kunming University of Science and Technology, Kunming 650500, China; 18206877260@163.com; 3 National Key Laboratory for Conservation and Utilization of Bio-Resources in Yunnan, Key Laboratory of Microbial Diversity in Southwest China, Ministry of Education, Yunnan Institute of Microbiology, School of Life Sciences, Yunnan University, Kunming 650091, China; lyp2019@mail.ynu.edu.cn

**Keywords:** *Docynia delavayi* (Franch.) Schneid., yeast biodiversity, fruit wine fermentation, non-*Saccharomyces* yeasts, aroma compounds

## Abstract

The community of epibiotic yeasts significantly influences the quality of *Docynia delavayi* (Franch.) Schneid. wine. The yeast diversity in four different *Docynia delavayi* (Franch.) Schneid. wines during the brewing stage was investigated using pure culture methods and high-throughput sequencing technology. A total of 229,381,292 sequencing bases were generated, yielding 323,820 valid sequences with an average length of 708 nt and identifying 93 operational taxonomic units (OTUs) from naturally fermented samples of *Docynia delavayi* (Franch.) Schneid. wine for classification purposes. At the early fermentation stage, *Hanseniaspora* sp. was identified as the dominant species, whereas at the late fermentation stage, *Hanseniaspora* sp., *Saccharomyces* sp., and *Candida californica* became predominant. From these samples, a total of 109 yeast strains were isolated from *Docynia delavayi* (Franch.) Schneid. wine. Three specific strains—LZX-76, LZX-89, and LZX-104—were further selected based on their growth characteristics along with hydrogen sulfide production, ester production, ethanol production, and tolerance levels. Through morphological examination and molecular biology techniques, these strains were identified as *Pichia fermentans* and *Hanseniaspora* spp. Additionally, a total of 29 volatile compounds were detected through simulated fermentation processes; these included 12 esters, 6 alcohols, 2 acids, 4 aldehydes, and 5 other compounds. When compared to commercial yeasts used as starters in winemaking processes, it was observed that utilizing yeast strains LZX-76, LZX-89, and LZX-104 resulted in an increased number of volatile compounds, which enhanced the aromatic profile characteristics of *Docynia delavayi* (Franch.) Schneid. wine by making its aroma richer and more complex. The findings from this study hold significant potential value for both the production practices and research endeavors related to *Docynia delavayi* (Franch.) Schneid. wine.

## 1. Introduction

**Figure 1:**
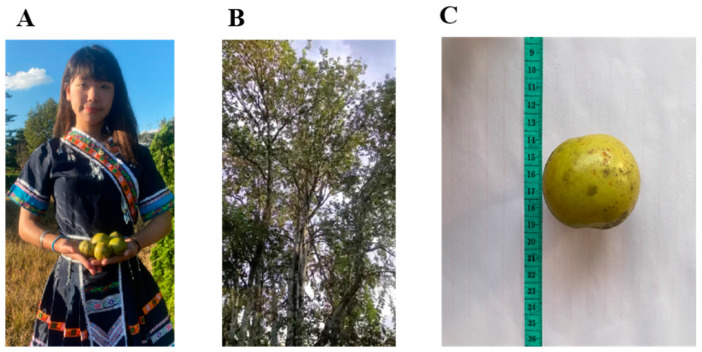
*Docynia delavayi* (Franch.) Schneid. It is a common medicinal and edible fruit for ethnic minorities in Yunnan Province. (**A**) *Docynia delavayi* (Franch.) Schneid., commonly eaten by the Hani people as a fruit or herb. (**B**) *Docynia delavayi* (Franch.) Schneid. The morphology of the whole tree. (**C**) Biological morphology of fresh fruit.

*Docynia delavayi* (Franch.) Schneid. is a tropical cash crop that belongs to the genus *Docynia*, which is situated within the *Maloideae* subfamily of the *Rosaceae* family (Figure 1). This species is primarily distributed across Asia, with significant production occurring in Yunnan, Guizhou, Sichuan, and other regions of southwest China [1]. *Docynia delavayi* (Franch.) Schneid. is recognized for its high nutritional value and serves both culinary and medicinal functions [2]. It contains an abundance of bioactive compounds, such as flavonoids, polyphenols, and saponins, which exhibit various beneficial activities, including antitumor effects, antimicrobial properties, immunomodulatory functions, anti-inflammatory responses, and regulation of cardiovascular system activity [3,4]. Furthermore, it also serves as an appetizer [5,6]. Currently, research on *Docynia delavayi* (Franch.) Schneid. primarily focuses on the functional components of its leaves and genetic breeding aspects. In contrast, studies concerning the deep processing of *Docynia delavayi* (Franch.) Schneid. fruits are relatively scarce [7,8,9]. Although the fruits of *Docynia delavayi* (Franch.) Schneid. have been developed for products such as fruit wine, dried fruit, juice, and vinegar, there is a lack of reported research on the dynamic changes in microbial communities during different fermentation stages of *Docynia delavayi* (Franch.) Schneid. wine.

Fruit wine is primarily made from fruit or juice (including pulp) as the main raw material. The beverage is generated through the fermentation of fruit juice, leading to a product with a specific alcohol content. Specifically, *Docynia delavayi* (Franch.) Schneid. has been utilized in this context. *Docynia delavayi* (Franch.) Schneid. is utilized in the production of wine characterized by its sweet and mellow flavor profile. Huang et al. [3] utilized commercial yeast obtained from Angel Yeast Co., Ltd. as the starter culture for fermenting *Docynia delavayi* (Franch.) Schneid. Optimization of the fermentation process was conducted using the response surface methodology based on single-factor experiments, evaluating parameters such as alcohol content, sensory scores, and soluble solid content. So, The research on microbial diversity during the fermentation process of *Docynia delavayi* (Franch.) Schneid. wine is currently insufficient, and the associated winemaking techniques are primarily derived from traditional practices [10,11,12,13,14,15]. The flavor substances in fruit wine are mainly formed during the growth and metabolism of yeast and other fungi, and there are more than 1000 kinds of them [16,17,18]. The precursor compounds of these flavor substances exist in the fruit as glycoside conjugates. Under the influence of yeast-related enzymes, these precursors dissociate into free flavor compounds, such as esters, alcohols, organic acids, aldehydes and ketones, sulfur-containing compounds, and terpenes, contributing to the unique flavor characteristics of fruit wines [19,20]. Li et al. [9] utilized local yeast to ferment *Korla pear* fruit wine and discovered that *Saccharomyces cerevisiae* X16 could modify the volatile aroma characteristics during fermentation, enhancing and complicating the overall aroma profile. Xu et al. [21] conducted a dynamic study on the microbial community and its volatile compounds throughout the natural fermentation process of *Prunus persica* ‘Compressa’ wine. Their findings indicated that *Kazachstania*, *Pichia*, *Aspergillus*, *Fructobacillus*, *Leuconostoc*, and *Lactobacillus* were dominant genera in the spontaneous fermentation of *Prunus persica* ‘Compressa’ wine and were closely associated with the production of 27 volatile compounds found in naturally fermented flat peach wine. However, *Docynia delavayi* (Franch.) Schneid. exhibits high acidity, which significantly impacts both microbial communities and metabolic processes [8]. Currently, there are no published reports regarding the microbial diversity associated with the natural fermentation of *Docynia delavayi* (Franch.) Schneid. wine. The complexity, diversity, and dynamic changes within microbial community structures during this fermentation process remain inadequately understood. Furthermore, no studies have reported on the succession patterns of microbial populations throughout the natural fermentation of *Docynia delavayi* (Franch.) Schneid. wine. Therefore, understanding how microbial diversity evolves during wine fermentation from *Docynia delavayi* (Franch.) Schneid. is crucial for enhancing both quality and flavor profiles in these wines.

In this study, we employed high-throughput sequencing to investigate the species composition of yeast and its dynamic changes. Culturable yeasts were also isolated using a pure culture method. The brewing characteristics of these culturable yeasts, including fermentation traits, tolerance to brewing conditions, and overall fermentation performance, were examined to assess their potential applications in the production of fruit wine from *Docynia delavayi* (Franch.) Schneid.

## 2. Materials and Methods

### 2.1. Sample Collection

*Docynia delavayi* (Franch.) Schneid. is a wild species found in Huize County, Yunnan Province, China. Fresh and ripe specimens of *Docynia delavayi* (Franch.) Schneid. were selected, cored, and squeezed to extract the juice. A total of 600 mL of *Docynia delavayi* (Franch.) Schneid. juice was prepared at a temperature of 28 °C. The juice underwent natural fermentation on a laboratory scale in a sterile 1000 mL flask, with the fermentation experiment being replicated three times under controlled conditions. The samples were fermented for varying durations: 1 day (DY1), 3 days (DY3), 5 days (DY5), and 15 days (DY15). Each prepared sample was divided into two portions; one portion was designated for the isolation of native yeast, while the other portion was utilized for DNA extraction and high-throughput sequencing.

### 2.2. DNA Extraction

Each sample comprised approximately 20 g of *Docynia delavayi* (Franch.) Schneid. wine, which was transferred into a 50 mL aseptic centrifuge tube. Subsequently, 20 mL of sterile water was added to the tube. The mixture was then shaken using a vortex oscillator (Pacific Biosciences, Menlo Park, CA, USA) for 15 min. Following this, the solution was filtered through a single layer of sterile gauze and centrifuged at 8000 rpm for 15 min at room temperature to collect the microorganisms. Total DNA was extracted from each sample utilizing the EZNA soil DNA kit (Omega Bio-tek; Norcross, GA, USA). This kit was procured from Majorbio BioPharm Technology Co., Ltd. (Shanghai, CHN) and employed in accordance with the manufacturer’s instructions. The concentration and purity of the extracted DNA were assessed using a NanoDrop 2000 spectrophotometer (Thermo Scientific, Wilmington, DE, USA). The purity of the DNA sample was assessed using the OD_260/280_ ratio, with values ranging from 1.8 to 2.0 deemed acceptable for subsequent experiments.

### 2.3. PCR Amplification and Illumina High-Throughput Sequencing

Polymerase chain reaction (PCR) amplification was performed to amplify the internal transcribed spacer (ITS) region using primers ITS3F (5′-GCATCGATGAAGAACGCAGC-3′) and ITS4R (5′-TCCTCCGCTTATTGATATGC-3′). (The primers were synthesized by Beijing Qingke Biotechnology Co., Ltd., Beijing, China) The PCR conditions were as follows: an initial denaturation at 75 °C for 5 min, followed by 35 cycles of denaturation at 95 °C for 30 s, annealing at 55 °C for 30 s, and elongation at 72 °C for 45 s. A final extension step was conducted at 72 °C for 10 min. (The enzyme was purchased from Beijing TransGen Biotech Co., Ltd. Beijing, China) PCR products from the same sample were pooled together and analyzed via a two-percent agarose gel electrophoresis. Subsequently, they were purified using AMPure^®^ PB magnetic beads (Tiangen Technology, Guangzhou, China). Raw sequence reads underwent demultiplexing, quality filtering, merging, and clustering into operational taxonomic units (OTUs), with a similarity cutoff of 97%. The species composition and differences were then analyzed utilizing the Majorbio Cloud Platform (https://cloud.majorbio.com).

### 2.4. Statistical Analysis

The analysis of Operational Taxonomic Units (OTUs) with 97% similarity was conducted using the Usearch software platform (version 11.0), available at http://drive5.com/uparse/ (accessed on 10 August 2024). The RDP classifier, employing a Bayesian algorithm or the BLAST (Basic Local Alignment Search Tool) comparison method, was utilized for taxonomic analysis of the OTU representative sequences exhibiting 97% similarity. At each taxonomic classification level—domain, kingdom, phylum, class, order, family, genus, and species—the community composition of each sample was assessed. The richness and diversity of the microbial community were evaluated through single-sample diversity (alpha diversity) analysis. OTUs or other taxonomic levels with 97% similarity were selected; subsequently, the alpha diversity index under various random samples was calculated using Mothur (version v.1.36.1). A dilution graph was generated utilizing R language tools. Based on the data table located in the tax_summary_a folder, a community bar graph was constructed using R language tools (version 4.0.1). To illustrate relationships among samples and species compositions visually, CIRcos-0.67-7 software (http://circos.ca/, accessed on 26 September 2024) was employed to create Circos plots that reflected both the proportions of dominant species within each sample (or group) and their distribution across different samples (groups). Additionally, UPGMA (Unweighted Pair-Group Method with Arithmetic Mean) clustering analysis was performed on the sample distance matrix to construct a hierarchical clustering tree representing sample-level relationships.

### 2.5. Determination of Yeast Growth Curves and Analysis of Fermentation Capacity

The size, color, dryness, viscosity, surface smoothness, and edge uniformity of yeast colonies on WL medium were utilized as criteria for preliminary identification. Subsequently, the yeast was further characterized through PCR amplification and sequencing of the 26S rDNA fragment. The selected yeast strain was inoculated into YPD liquid medium and cultured at 180 rpm at 28 °C for a duration of 24 h. The optical density (OD) of the sterile YPD liquid medium was measured every 2 h at a wavelength of 600 nm.

A 2% yeast suspension was inoculated into YPD liquid medium containing Durham tubes and incubated at 28 °C for 48 h. The formation of bubbles in the Durham tubes was observed. Positive reactions were recorded as “+” if bubbles formed, while negative reactions were indicated as “−” [17,22].

### 2.6. Molecular Biological Identification of Yeast

The yeast genome was extracted using a microwave oven as a template [23,24]. Universal primers NL1 (5′-GCATATCAATAAGCGGAGGAAAAG-3′) and NL4 (5′-GGTCCGTGTTTCAAGACGG-3′) were employed for amplification. The 26S rDNA D1/D2 region was amplified via polymerase chain reaction (PCR). The resulting PCR products were sequenced by Kunming Qingke Biotechnology Co., Ltd. (Kunming, China) DNA sequence alignment was performed using the Basic Local Alignment Search Tool provided by the National Center for Biotechnology Information, MD, to identify the yeast species. The Neighbor-Joining method in MEGA software (version X)was utilized to construct the phylogenetic tree [25], and suitable strains were selected for subsequent experiments based on experimental outcomes and identification results.

### 2.7. Capacity of Yeast to Produce H_2_S, Ethanol, and Ester

Each 5 μL activated yeast suspension sample was absorbed into a bismuth, glucose, and glycine yeast agar medium (BIGGY) (1 L), which consisted of 10 g of glucose, 1 g of yeast extract powder, 5 g of ammonium citrate, 3 g of sodium sulfite, 10 g of ammonium sulfite, and 15 g of agar at a pH of 6.8 ± 0.2. The mixture was boiled and sterilized for one minute. Once the yeast suspension was fully incorporated into the medium, it was sealed with a sealing film and incubated inversely at 28 °C for 5 days to observe colony coloration. A darker color indicates a stronger ability to produce hydrogen sulfide; the commercial yeast CEGC (Angel Yeast Co., Ltd. Wuhan, China, *Saccharomyces cerevisiae*) was used as a control [26].

A pipette was used to transfer 10 μL of the activated yeast suspension into the submedium containing 2,3,5-triphenyltetrazolium chloride (TTC) (1 L), which consisted of TTC (0.5 g), glucose (5 g), and agar (15 g). This mixture was then boiled for 2 min to ensure sterilization. After adding the TTC to the lower medium, the yeast suspension was fully absorbed into it. The sealed culture was then incubated at 30 °C for 48 h, ensuring that the upper medium containing MgSO_4_ (0.4 g), KH_2_PO_4_ (1 g), yeast extract (1.5 g), peptone (2 g), glucose (10 g at 1%), and agar (20 g) was completely covered. After a dark incubation period of 36 h, sufficient colonies were observed and recorded as an indicator of ethanol production capacity in the yeast strains [27]. Each strain was tested three times, with the commercial yeast CEGC serving as a control.

A sample of 10 µL of activated yeast suspension was introduced into the YPD ester production qualitative screening medium (1 L), which consisted of 50 g glucose, 20 g peptone, 10 g yeast extract powder, 15 g agar, and 4 g tributyrin, to assess ester production. Following complete absorption of the yeast suspension by the medium, it was sealed with a film and incubated inversely at 28 °C for 4 days. The colony density surrounding the transparent circle was then observed and measured [28]. Additionally, the commercial yeast CEGC was inoculated as a control.

### 2.8. Analysis of Yeast Tolerance to Abiotic Factors

The non-*Saccharomyces* were inoculated at a concentration of 2% in YPD liquid medium, with varying ethanol volume fractions (3%, 6%, 9%, 12%, and 15% *v*/*v*), SO_2_ volume fractions (60, 180, 300, and 360 mg/L), glucose mass concentrations (100, 150, 200, 250, and 300 g/L), and temperatures (4 °C, 20 °C, 30 °C, and 40 °C). The commercial yeast CEGC was incubated at a temperature of 28 °C for a duration of 24 h while being agitated at a speed of 180 rpm. The optical density (OD) was measured at a wavelength of 600 nm; each treatment was replicated three times.

### 2.9. Docynia delavayi (Franch.) Schneid. Simulated Fermentation of Wine

Fresh, ripe, and unspoiled *Docynia delavayi* (Franch.) Schneid. juice was extracted using a juicer (Midea, WJE2802D, Kunming, China). Potassium pyrosulfite (100 mg/L), diammonium hydrogen phosphate (DAP) (400 mg/L), and pectinase (20 mg/L) were added to the filtrate and incubated at room temperature for 12 h. Glucose was then added to adjust the sugar content to 24°Brix. The control group was inoculated with the commercial yeast CEGC. Native non-*Saccharomyces* strains (LZX-89, LZX-104, and LZX-109) were inoculated into the fermentation solution at a concentration of 2%, followed by static fermentation conducted at 20 °C.

### 2.10. Docynia delavayi (Franch.) Schneid. Volatile Components and Sensory Evaluation of Wine

After fermentation, the *Docynia delavayi* (Franch.) Schneid. samples were prepared. The supernatant obtained from centrifugation at 3000× *g* for 10 min was utilized for the analysis of volatile components and sensory evaluation. The total sugar content, total acidity, and pH of the *Docynia delavayi* (Franch.) Schneid. wines were assessed using Fehling titration, acid–base neutralization methods, and a pH meter [29]. Volatile compounds were isolated and identified using an Agilent 7890B Gas Chromatograph coupled with a 5975B Mass Spectrometer, employing HP-INNOWAX capillary columns (J&W Scientific, Folsom, CA, USA) as per standard procedures. The inlet temperature was maintained at 250 °C with a splitless injection mode. High-purity helium gas (>99.999%; Sichuan WinTec Specialty Gas Company, Chengdu, China) served as the carrier gas at a flow rate of 1 mL/min. The oven temperature program commenced at 50 °C for one minute before ramping to 220 °C at a rate of 3 °C/min and holding this temperature for 5 min. The temperatures for the transfer line heater, ion source, and quadrupole were set to 250 °C, 250 °C, and 150 °C, respectively. In full-scan mode (*m*/*z* range: 30–350), electron ionization (EI) was conducted at an energy level of 70 eV. To calculate the total content of each volatile compound, references [30,31] were consulted. The sensory evaluation of *Docynia delavayi* (Franch.) Schneid. wine was conducted by a panel of 10 professional wine tasters, which included no fewer than 5 first-level tasters. The assessment utilized standard aroma characteristics to describe the sensory profile of the wine. These standard aroma characteristics were based on the design principles outlined in the French wine aroma product “wine nose”. For this evaluation, ISO-standard crystal wine glasses were employed to hold 30 mL samples of the wine at a controlled room temperature ranging from 20 to 25 °C. Each taster initially smelled the still wine sample for a duration of 5–8 s before swirling the glass for an additional 5–10 s. Subsequently, they described the aromatic attributes of each sample using eight characteristic terms: acidity, sweetness, color, bitterness, aftertaste, richness, astringency, and clarity [22,32].

### 2.11. Data Analysis

The data are presented as means ± standard deviations. Statistical analysis was conducted using SPSS version 21.0 to assess significant differences between the groups. A *p*-value of less than 0.05 was considered statistically significant, and the data were plotted using Origin 2022 software. Each experiment was performed in triplicate to ensure the reliability of the results.

## 3. Results and Discussion

### 3.1. Determination of Yeast Biodiversity Using High-Throughput Sequencing Methods

A total of 229,381,292 sequencing bases and 323,820 valid sequences with an average length of 708 nucleotides were obtained from the natural fermentation samples of *Docynia delavayi* (Franch.) Schneid. wine, exhibiting a similarity of 97%. Subsequently, coverage analysis was conducted to assess the representation of low-abundance operational taxonomic units (OTUs) within the samples. The results indicated that the coverage for all samples was 1.00, demonstrating that this study comprehensively captured low-abundance OTUs. This sequencing effort accurately reflected the true microbial populations present.

**Figure 2:**
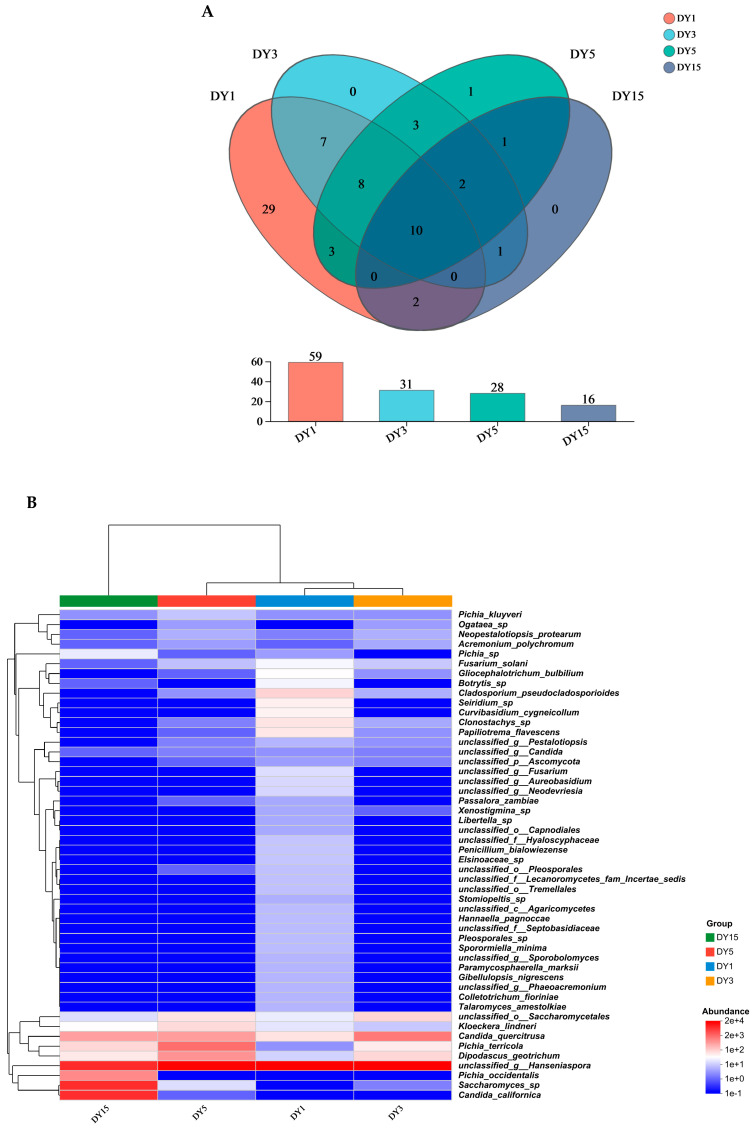

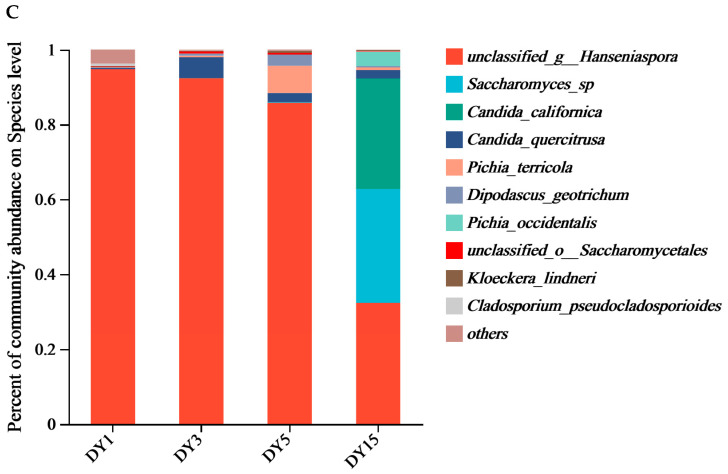
*Docynia delavayi* (Franch.) Schneid. Biodiversity of and dynamic changes in wine fungi. (**A**) Fungal community distribution at different stages of natural fermentation. (**B**) Community heat map analysis at the species level. (**C**) Relative abundance and dynamic changes in different fungal communities in natural fermentation stages.

A total of 134 fungi were isolated through natural fermentation using *Docynia delavayi* (Franch.) Schneid. wine. Specifically, the samples DY1, DY3, DY5, and DY15 yielded 59, 31, 28, and 16 fungal species, respectively (Figure 2A).

Figure 2B presents the results of the cluster heat map for the top 50 fungal species. High species abundances in the samples are represented in red, while low species abundances are depicted in blue. Samples DY1 and DY5 were grouped within a clade, indicating a similar species composition between these two samples. In contrast, samples DY3 and DY15 clustered together on a separate branch. The predominant fungi detected included various yeasts, such as *Pichia kluyveri*, *Kloeckera lindneri*, *Candida quercitrusa*, *Pichia terricola*, *Candida californica*, and *Pichia occidentalis*. Additionally, several pathogenic bacteria were identified; these included *Fusarium solani*, *Cladosporium cladosporioides*, *Mycosphaerella marksii*, *Colletotrichum fioriniae*, *Dipodascus geotrichum*, and *Gibellulopsis nigrescens*.

The strains observed on day 1 (DY1), day 3 (DY3), and day 5 (DY5) of the natural fermentation were predominantly single strains, with *Hanseniaspora* sp. being the dominant yeast species, comprising 94.75%, 92.34%, and 85.80% of the total strains, respectively (Figure 2C). As fermentation progressed, there was a rapid increase in microbial diversity by DY15, where the predominant species included *Hanseniaspora* sp. (32.30%), *Saccharomyces* sp. (30.42%), and *Candida californica* (29.56%). The obtained diversity index results further corroborated changes in species diversity throughout the fermentation (Table S1). Notably, the Shannon index reached its peak value at DY15; however, it exhibited a decline during DY3 and DY5, ultimately recording its lowest value at DY1.

### 3.2. The Diversity of Yeast Determined by the Pure Culture Method

A total of 109 indigenous yeast strains were isolated from four stages of the natural fermentation process of *Docynia delavayi* (Franch.) Schneid. wine. Based on the morphological characteristics observed in colonies grown on WL nutrient agar, 15 yeast strains were initially selected for further study. These strains are designated as LZX-5, LZX-9, LZX-23, LZX-26, LZX-37, LZX-51, LZX-75, LZX-76, LZX-81, LZX-89 (note: this strain is mentioned twice), LZX-94, LZX-95, LZX-99, LZX-104, and LZX-109. The morphology of their colonies and cells is summarized in Table 1 (A). The colony surface of strain LZX-5 was smooth with milky white edges and a green center. Strain LZX-9 exhibited a flat but uneven colony with milky white edges and a green center. The colony protrusion of strain LZX-23 was irregularly shaped; it had white margins and a light-blue center. Strain LZX-37 presented a flat yet rough appearance with white edges transitioning from green to light blue at the center. Strain LZX-51 displayed a flat and smooth colony characterized by creamy white edges surrounding a light-green center. Similarly, LZX-75 also had a flat and smooth structure with creamy white edges adorned by light-blue markings. In contrast, LZX-76’s colony was flat but rough-edged with light-green centers surrounded by white margins. The colony morphology for strain LZX-81 appeared flat and rough in texture while producing bubbles during growth. Strain LZX-89 featured an entirely white coloration that was both flat and smooth, whereas LZX-94 showcased milky white protrusions that were also smooth. Strain LZX-95 possessed smoothly protruding features along its margin, which was distinctly white against its light-green central area. LZX-99 demonstrated an overall appearance that was both flat yet rough while maintaining whiteness throughout its structure. For strain LZX-104, the protrusions were slightly smoother than the others’; it exhibited light-green edging leading into shades ranging from green to blue at the core. Finally, LZX-109 had an overall whitish hue combined with being both flat yet rugged and featuring lighter-blue centers. In terms of cellular morphology, strains LZX-51 and LZX-76 were cylindrical in shape, while strains LZX-37 and LZX-99 had round-shaped cells. The remaining strains predominantly exhibited oval cell shapes.

**Figure 3:**
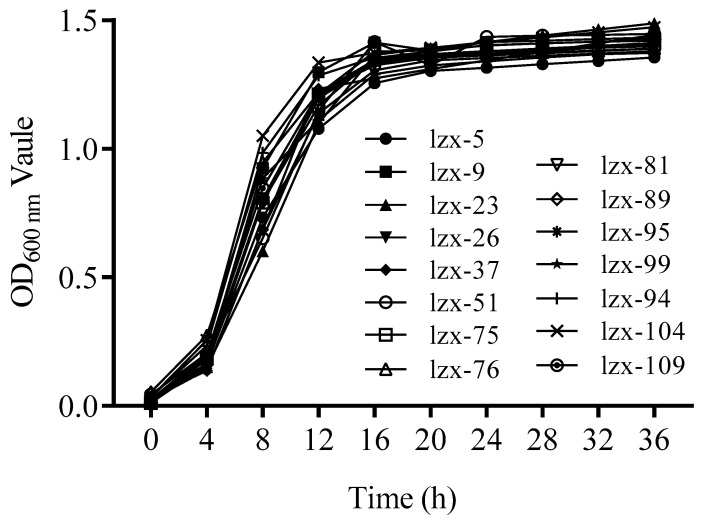
Growth curves of the yeasts isolated from *Docynia delavayi* (Franch.) Schneid. wine.

The growth curves of the strains are illustrated in Figure 3. The period from 0 to 4 h represents a lag phase, while the interval from 4 to 18 h corresponds to a logarithmic growth phase. Beyond 18 h, the cultures entered a stable growth phase. The fermentation capacity is detailed in Table 1 (B), which indicates that LZX-23 produced less gas during fermentation, suggesting potential turbidity issues and an absence of brewing advantages [33]. In contrast, LZX-51, LZX-75, and LZX-94 exhibited robust fermentation capabilities, with LZX-76 demonstrating the strongest fermentation ability among them.

**Figure 4:**
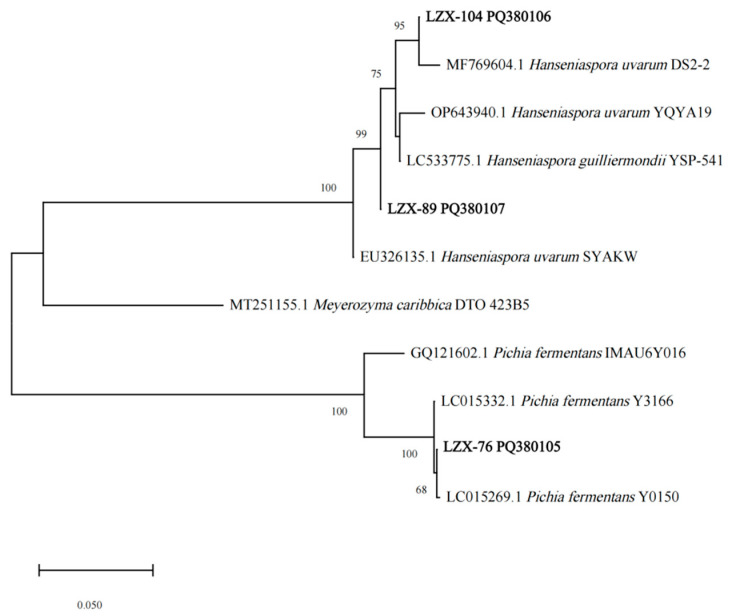
Phylogenetic tree of yeast strains based on 26S rDNA sequences.

By molecular biological identification, it was determined that strains LZX-9, LZX-95, LZX-26, LZX-89, and LZX-75 exhibited 100% similarity to *Hanseniaspora pseudoguilliermondii* in the GenBank database. Additionally, strains LZX-78 and LZX-104 showed a 100% similarity with *Hanseniaspora uvarum*. Strains LZX-23, LZX-51, LZX-76, LZX-99, and LZX-109 were found to be 100% similar to *Pichia fermentans* (Table 2). In summary, strains LZX-76, LZX-89, and LZX-104 were selected for subsequent experiments. These strains have been submitted to the NCBI database under accession numbers PQ380105, PQ380107, and PQ380106, respectively. The constructed phylogenetic tree is illustrated in Figure 4. Strain LZX-76 (PQ380105) clustered with LC015269.1 *Pichia fermentans* Y0150, LC015332.1 *Pichia fermentans* Y3166, and GQ121602.1 *Pichia fermentans* IMAU6Y016 within a single branch. Meanwhile, strains LZX-89 (PQ380107) and LZX-104 (PQ380106) formed a distinct branch alongside *Hanseniaspora* species, suggesting that strain L ZX-76 (PQ380105) belongs to *Pichia fermentans*, while strains LZX-89 (PQ380107) and LZX-104 (PQ380106) were classified under *Hanseniaspora*.

### 3.3. Analysis of the H_2_S Production, Ethanol Production, and Ester Production Capacity of the Yeasts

The glycine present in BIGGY culture medium has the potential to inhibit the growth of certain bacteria. Additionally, bismuth sulfite can be formed through the reaction between bismuth ammonium citrate and sodium sulfite; this compound appears brown or black with a metallic luster and is effective in inhibiting the growth of most bacterial species [34]. Non-*Saccharomyces* can also produce hydrogen sulfide (H_2_S) via sulfate reduction while simultaneously enhancing the aroma profile of wine. However, high concentrations of H_2_S emit an unpleasant odor reminiscent of rotten eggs, which detracts from the sensory quality of fruit wines. BIGGY agar medium was employed to assess the yeasts’ capacity for H_2_S production. In this context, darker-colored colonies indicated higher levels of H_2_S production, whereas lighter-colored colonies suggested lower levels. Specifically, white colonies were indicative of no H_2_S production [26,33]. The results demonstrated that strains CEGC and LZX-76 exhibited white coloration, indicating they did not produce H_2_S. Conversely, strains LZX-89 and LZX-104 displayed brown coloration, signifying their ability to produce H_2_S (Figure S1A).

Yeast plays a crucial role in the production of ethanol and carbon dioxide by metabolizing sugars present in cider, a fundamental reaction in the process of cider brewing. During this fermentation process, yeast converts glucose into ethanol and carbon dioxide while simultaneously releasing a small amount of energy. This biochemical transformation not only results in the creation of an alcoholic beverage but also contributes to the distinctive aroma and flavor profile characteristic of fruit wines. The principle behind color development in TTC relies on the reduction of dehydrogenase, which leads to the formation of red or purple 1,3,5-triphenylmethyl (TPF) compounds and precipitated cells. This process results in cell coloration that can appear dark red, pink, slightly red, or colorless. Notably, enzyme activity is positively correlated with the intensity of red coloration observed in colonies. Consequently, one can assess a strain’s ethanol production capacity by examining the darkness of its colony [35,36]. The color rendering results are illustrated in Figure S1B. Strain CEGC exhibited a deep red hue. Strain LZX-104 displayed a reddish edge with a pale pink center. Strain LZX-89 appeared pink with a lighter pinkish-white core. In contrast, strain LZX-76 was characterized as pale pink. Among these strains, CEGC demonstrated the highest ethanol production capacity while LZX-76 showed the lowest.

Ester is an essential compound found in various fermented wines. The esterase produced by the metabolism of esterogenic strains can either decompose or synthesize butyrate triglycerides. A transparent circle will manifest on the solid medium, indicating ester production activity. The higher the ratio, the greater the capacity for ester production; conversely, a lower ratio indicates diminished capacity [37,38]. As illustrated in Table 3, strain LZX-89 exhibited the highest D/d value (1.72) and demonstrated superior ester production capability. In contrast, strain CEGC recorded the lowest D/d value (1.38), reflecting its having the weakest ester production capacity.

### 3.4. Tolerance of Yeast to Abiotic Factors

To evaluate the tolerance of the selected yeast strains to brewing conditions, we cultured all test strains under varying concentrations of ethanol, glucose, and sulfur dioxide (SO_2_) and different temperature treatments. Optical density (OD) values were measured at a wavelength of 600 nm, with the commercial yeast CEGC serving as the control.

**Figure 5:**
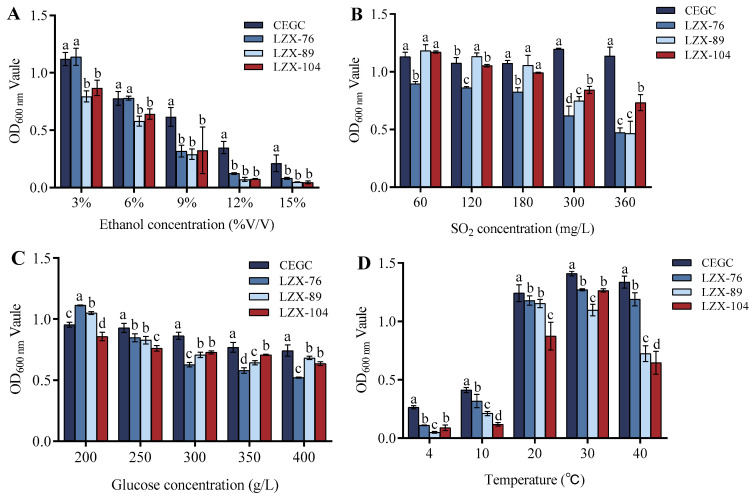
Properties of oenological condition tolerances of the selected yeasts isolated from *Docynia delavayi* (Franch.) Schneid. wine: (**A**) ethanol tolerance, (**B**) SO_2_ tolerance, (**C**) glucose tolerance, and (**D**) temperature tolerance. Different lowercase letters above the standard deviation bars indicate significant differences (*p* < 0.05).

Ethanol serves as the primary fermentation substrate for yeast; however, excessively high concentrations can inhibit yeast growth. Most non-*Saccharomyces* strains exhibit lower tolerance to extreme environments, such as elevated ethanol levels, compared to *Saccharomyces cerevisiae*. This difference often results in a reduced ethanol concentration during the fermentation process. Maintaining a moderate ethanol level can enhance yeast survival rates and promote the release of additional enzymes or esters [22,39]. In this study, an analysis of the ethanol tolerance among four yeast strains revealed that their growth significantly declined with increasing ethanol volume fractions when compared to strain CEGC. Notably, strain LZX-76 demonstrated higher ethanol tolerance than both strains LZX-89 and LZX-104; thus, strains CEGC and LZX-76 exhibited superior resistance to ethanol (Figure 5A).

A specific amount of SO_2_ is typically added during the brewing process of fruit wine to inhibit the growth of various bacteria and to prevent the aging of the wine [40]. The tolerance levels of different yeast strains to SO_2_ are illustrated in Figure 5B. When the concentration of SO_2_ ranged from 60 mg/L to 300 mg/L, all strains exhibited insensitivity to changes in the mass concentration of SO_2_. Notably, at a mass concentration of 300 mg/L, CEGC and LZX-104 demonstrated higher tolerance to SO_2_ compared to LZX-76 and LZX-89.

In the brewing process of fruit wine, sugar serves as a carbon source for fermentation and promotes the growth and metabolic activities of microbiota. However, high concentrations of sugar can inhibit yeast growth, leading to reduced glucose metabolism. Additionally, elevated osmotic pressure may cause cellular water loss, further diminishing cell activity. Therefore, it is essential to investigate the glucose tolerance of various yeast strains [41,42]. The results are presented in Figure 5C. All selected strains exhibited robust growth at the tested glucose concentrations (200–400 g/L), with strains CEGC, LZX-89, and LZX-104 demonstrating significantly higher tolerance than strain LZX-76 at elevated glucose levels of 400 g/L. The temperature tolerance of different yeast strains is illustrated in Figure 5D. All strains displayed poor tolerance at temperatures of 4 °C and 10 °C. Optimal tolerance was observed at temperatures of 20 °C and 30 °C. At 40 °C, strains LZX-89 and LZX-104 showed diminished tolerance; conversely, strains CEGC and LZX-76 exhibited strong resilience under these conditions.

### 3.5. Brewing Properties of Yeast

To assess the fermentation performance of local non-*Saccharomyces* strains, wine was produced through laboratory fermentation using *Docynia delavayi* (Franch.) Schneid. Table 4 presents the fundamental physical and chemical parameters of *Docynia delavayi* (Franch.) Schneid. wine fermented with various yeast strains. The residual sugar content for LZX-104 was measured at 153.22 mg/L, while LZX-89 exhibited a range of approximately 166.17 mg/L. Notably, the residual sugar content and total acidity of LZX-104 were significantly higher than those observed for the other strains. The analysis included four samples of *Docynia delavayi* (Franch.) Schneid. at pH levels ranging from 3.6 to 3.7, revealing no significant differences among the wines tested. However, it is important to note that the total acidity of the LZX-104 sample was markedly greater than that found in the LZX-76, LZX-89, and CEGC samples.

**Figure 6:**
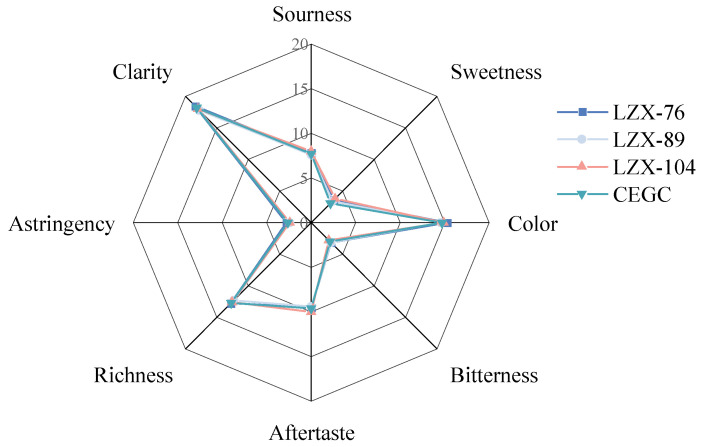
Radar chart of sensory characteristics of wine fermented by strains of *Docynia delavayi* (Franch.) Schneid.

To investigate the fermentation process of *Docynia delavayi* (Franch.) Schneid. wine, a sensory analysis was conducted on wines produced from this species, utilizing various yeast strains. The sensory characteristics of the resulting wines were comprehensively evaluated. This study examined three distinct varieties of *Docynia delavayi* (Franch.) Schneid. wine. However, no significant differences were observed in the sensory attributes of the wines, including sourness, sweetness, color, bitterness, aftertaste, richness, astringency, and clarity (Figure 6).

### 3.6. Volatile Aroma Characteristics of Docynia delavayi (Franch.) Schneid. Wine

Studies have demonstrated that utilizing local yeasts for fermentation significantly enhances the flavor diversity and typicity of wine regions, leading to more complex and aromatic characteristics in fruit wines. This ultimately improves the overall quality of flavor [9]. The fermentation process of *Docynia delavayi* (Franch.) Schneid. with selected yeast strains was further investigated through GC-MS analysis, focusing on the volatile aroma characteristics of the resulting wine (Table 5). A total of 29 volatile compounds were identified in *Docynia delavayi* (Franch.) Schneid. wine, comprising 12 esters, 6 alcohols, 2 acids, 4 aldehydes, and 5 other compounds. Notably, a greater variety of volatile compounds—23 in total—was detected in the wine produced from LZX-76-fermented *Docynia delavayi* (Franch.) Schneid. In contrast, the wine fermented using CEGC exhibited a lesser variety, with only 18 distinct compounds. Specific compounds such as 6-methyl-5-hepten-2-one, geranylacetone, and cyclododecan were exclusively found in LZX-76-fermented wine; conversely, phenylprop-2-enoate was uniquely present in CEGC-fermented samples. The use of the selected yeast strains for fermenting *Docynia delavayi* (Franch.) Schneid. resulted in an increased quantity of various other volatile compounds compared to employing CEGC alone as a starter culture.

A total of 11 esters were identified in the LZX-104-fermented *Docynia delavayi* (Franch.) Schneid. wine. In contrast, 10 esters were detected in the wines fermented with LZX-76 and LZX-89. Additionally, eight esters were identified in the CEGC-fermented *Docynia delavayi* (Franch.) Schneid. wine (Table 5). The concentration of esters across different samples varied, ranging from 24.67 mg/L for LZX-76 to 9.69 mg/L for LZX-89. Ethyl ester compounds, such as ethyl acetate, ethyl butyrate, and ethyl caproate, predominated in all the samples; notably, ethyl butyrate was the dominant ester during fermentation with LZX-89. Conversely, both ethyl caproate and ethyl butyrate emerged as dominant esters in wines fermented with LZX-76 and LZX-104. Regarding alcohol content, five distinct alcohols were identified across the three wine samples; four of these were present in the fermentations involving LZX-89 and LZX-104 (Table 5). The alcohol content of wines produced using LZX-76 was comparable to that of those made with LZX-89, both being higher than that observed for wines fermented with LZX-104. Ethanol and hexene-1 alcohols constituted the primary alcoholic components found in all *Docynia delavayi* (Franch.) Schneid. wines; however, most alcohols were consistently detected across all samples, except for 5-hexene-1 alcohols. Two acids—acetic acid and methylbutyric acid—were identified within all samples analyzed; however, methylbutyric acid was absent from the *Docynia delavayi* (Franch.) Schneid. wine fermented with LZX-76. Notably, the highest concentration of acetic acid was observed in this sample. The detected aldehydes and ketones were primarily benzaldehyde and trans-2-hexenal, with benzaldehyde predominantly present in the *Docynia delavayi* (Franch.) Schneid. wine produced through LZX-76 fermentation. In contrast, trans-2-hexenal was mainly identified in CEGC’s *Docynia delavayi* (Franch.) Schneid. wine. Significant differences were noted in the contents of aldehydes and ketones across various fermentation treatments. Furthermore, four additional compounds were identified; however, the LZX-104-fermented *Docynia delavayi* (Franch.) Schneid. wine contained only two of these compounds, representing the lowest quantity among all samples analyzed. The concentrations of these compounds reached their peak in LZX-76-fermented *Docynia delavayi* (Franch.) Schneid. wine, while being at their lowest levels in CEGC-fermented *Docynia delavayi* (Franch.) Schneid. wine. These findings underscore the variability in the volatile characteristics of *Docynia delavayi* (Franch.) Schneid. wine, which is influenced by different yeast strains used as starter cultures.

## 4. Conclusions

To the best of our knowledge, this study represents the first systematic analysis of the biodiversity and winemaking characteristics of yeasts native to *Docynia delavayi* (Franch.) Schneid. wine. A high-throughput sequencing method was employed to detect a diverse array of yeast species. Additionally, three types of culturable yeasts were isolated using traditional purification techniques. The growth characteristics, fermentation performance, ethanol production, ester production, and hydrogen sulfide production exhibited notable differences among these native yeasts. The volatile aromatic compounds present in fermented *Docynia delavayi* (Franch.) Schneid. wine, including ethyl acetate and ethyl butanoate, contribute to variations in aroma substances. These modifications significantly enrich the flavor profile and complexity of *Docynia delavayi* (Franch.) Schneid. Wine. Consequently, these native yeast inoculations hold significant potential for application in the production of *Docynia delavayi* (Franch.) Schneid. wine.

## Figures and Tables

**Table 1:** Colony morphotypes of indigenous wine yeasts of *D. delavayi* on WL nutrient agar (A) and in the fermentation ability test (B).

(A)				(B)			
Strain Number	Colony Color		Colony Topography	12 h	24 h	36 h	48 h
LZX-5	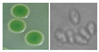	White on the surface and dark green in the center	Convex, spherical, smooth, opaque surface	+	++	++	+
LZX-9	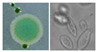	Light green on the surface around and green in the center	Convex, wrinkled surface, irregular edge	++	++	++	++
LZX-23	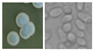	White on the surface and blue in the center	Convex, opaque surface, irregular edge	−	+	+	+
LZX-26	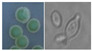	White on the surface and green and blue in the center		+	++	++	++
LZX-37	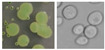	Tender green	Convex, spherical, smooth, opaque surface	+	++	++	+
LZX-51	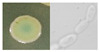	Milky on the surface and blue in the center		−	+++	+++	+++
LZX-75	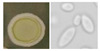	White on the around and light green in the center	Convex, wrinkled surface, irregular edge	++	+++	+++	+++
LZX-76	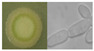	Light green		+++	+++	+++	+++
LZX-81	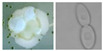	White	Convex, wrinkled surface, irregular edge	+	++	++	+
LZX-89	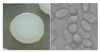	White	Convex, opaque surface, irregular edge	++	++	++	++
LZX-94	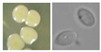	Slightly yellow to cream	Convex, spherical, smooth, opaque surface	+	+++	+++	++
LZX-95	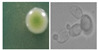	White on the surface and cyan in the center		++	++	++	+
LZX-99	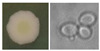	Milky on the around and light green in the center	Convex, wrinkled surface, irregular edge	++	++	++	++
LZX-104	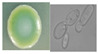	White on the surface and green and blue in the center	Convex, spherical, smooth, opaque surface	+	++	++	++
LZX-109	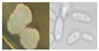	Milky on the surface and light blue in the center	Convex, wrinkled surface, irregular edge	+	++	++	++

Note: +, ++, +++ respectively gas accounted for a quarter, half, all, − said no.

**Table 2:** Strain comparison results.

Strains	PCR Fragment Length	Login ID	Similarity	Strain Species
LZX-104	571–588 bp	OQ 305050.1	100%	*Hanseniaspora uvarum*
LZX-89	584–591 bp	MH 465057.1	100%	*Hanseniaspora pseudoguilliermondii*
LZX-76	571–631 bp	MT 036025.1	100%	*Pichia fermentans*

**Table 3:** Lipid production screening of the four yeast strains.

Strains	Strain Transparent Circle Diameter (D)/cm	Colony Diameter (d)/cm	D/d Value
CEGC	0.72 ± 0.03	0.52 ± 0.03	1.38
LZX-76	1.67 ± 0.06	1.09 ± 0.04	1.53
LZX-89	1.75 ± 0.02	1.05 ± 0.01	1.72
LZX-104	1.57 ± 0.01	1.08 ± 0.04	1.45

**Table 4:** Basic physical and chemical parameters.

Strains	Residual Sugar (mg/L)	Total Acidity (mg/g)	pH
CEGC	155.39 ± 0.49 ^b^	10.37 ± 0.10 ^b^	3.64 ± 0.01 ^a^
LZX-89	153.22 ± 0.85 ^c^	10.30 ± 0.16 ^b^	3.70 ± 0.01 ^a^
LZX-104	166.17 ± 0.76 ^a^	11.09 ± 0.05 ^a^	3.64 ± 0.01 ^a^
LZX-109	164.91 ± 0.63 ^a^	10.34 ± 0.08 ^b^	3.66 ± 0.02 ^a^

Note: Values in the same column with different lowercase letters are significantly different (*p* < 0.05).

**Table 5:** Volatile compounds (mg/L) in *Docynia delavayi* (Franch.) Schneid. wines fermented with different yeasts.

No.	Compounds	CAS	CEGC	LZX-76	LZX-89	LZX-104
1	Methyl butanoate	623-42-7	0.38 ± 0 ^d^	0.67 ± 0.02 ^a^	0.47 ± 0.02 ^c^	0.58 ± 0.01 ^b^
2	Ethyl hexanoate	123-66-0	5.57 ± 0.99 ^b^	7.78 ± 0.15 ^a^	0.42 ± 0.04 ^c^	8.3 ± 0.24 ^a^
3	Butyl butanoate	109-21-7	0.59 ± 0.02 ^b^	/	/	0.78 ± 0.11 ^a^
4	Ethyl pentanoate	539-82-2	/	0.39 ± 0.01 ^a^	/	0.36 ± 0.01 ^b^
5	Methyl hexanoate	106-70-7	/	0.45 ± 0.02 ^b^	0.4 ± 0.01 ^c^	0.57 ± 0.04 ^a^
6	butyl acetate	123-86-4	0.83 ± 0.01 ^d^	0.95 ± 0.02 ^c^	1.17 ± 0	1.38 ± 0.02 ^a^
7	Ethyl butanoate	105-54-4	5.6 ± 0.26 ^c^	6.55 ± 0.4 ^b^	4.36 ± 0.09 ^d^	7.59 ± 0.18 ^a^
8	Ethyl propanoate	105-37-3	0.35 ± 0.02 ^d^	0.79 ± 0.02 ^a^	0.47 ± 0.02 ^c^	0.55 ± 0.02 ^b^
9	Ethyl acetate	141-78-6	3.44 ± 0.07 ^a^	2.94 ± 0 ^b^	2.79 ± 0.01 ^b^	2.54 ± 0.2 ^d^
10	2,2,4-trimethyl-1,3-pentandio disobutyrate	6846-50-0	/	0.73 ± 0.01 ^a^	0.52 ± 0.05 ^c^	0.63 ± 0.01 ^b^
11	Diisobutyl phthalate	84-69-5	/	3.37 ± 9.02 ^a^	0.83 ± 0.01 ^c^	1.17 ± 0.02 ^b^
12	Ethyl (2E)-3-phenylprop-2-enoate	4192-77-2	0.69 ± 0.11	/	/	/
	Σ Esters		17.46 ± 1.12 ^c^	24.61 ± 12.02 ^a^	9.69 ± 0.13 ^d^	21.72 ± 0.33 ^b^
13	4-methyl-2-pentanol	928-95-0	0.64 ± 0.03 ^a^	0.56 ± 0.01 ^b^	0.5 ± 0.01 ^c^	0.47 ± 0.01 ^c^
14	2-ethylhexanol	104-76-7	0.41 ± 0.01 ^b^	0.62 ± 0.09 ^a^	0.38 ± 0.01 ^b^	0.35 ± 0.01 ^b^
15	(E)-2-hexen-1-ol	111-27-3	5.06 ± 0.1 ^a^	4.45 ± 0.16 ^c^	4.73 ± 0.05 ^b^	3.81 ± 0.13 ^d^
16	Hexan-1-ol	108-11-2	36.16 ± 0.07 ^a^	32.13 ± 0.64 ^b^	34.32 ± 0.74 ^ab^	29.02 ± 2.15 ^c^
17	5-hexen-1-ol	821-41-0	0.35 ± 0.08 ^b^	0.74 ± 0 ^a^	/	/
18	Ethanol	64-17-5	28.2 ± 0.08 ^b^	28.9 ± 0.1 ^a^	26.74 ± 0.05 ^c^	25.75 ± 0.04 ^d^
	∑Alcohols		70.82 ± 0.27 ^a^	67.41 ± 19.02 ^b^	66.67 ± 0.71 ^b^	59.4 ± 2.18 ^c^
19	Trans-2-hexenal	6728-26-3	1.65 ± 0.02 ^a^	1.38 ± 0.01 ^b^	0.92 ± 0.05 ^c^	/
20	6-methyl-5-hepten-2-one	110-93-0	/	0.34 ± 0	/	/
21	Benzaldehyde	100-52-7	8.06 ± 0.43 ^b^	10.38 ± 0.16 ^a^	9.45 ± 0.34 ^a^	6.29 ± 0.62 ^c^
22	Hexanal	66-25-1	/	0.65 ± 0.01 ^b^	0.75 ± 0.02 ^a^	0.54 ± 0 ^c^
23	Geranylacetone	3796-70-1	/	0.56 ± 0	/	/
	∑Aldoketones		9.44 ± 0.44 ^c^	13.59 ± 25.02 ^a^	11.11 ± 0.3 ^b^	6.83 ± 0.62 ^d^
24	2-methylbutanoic acid	116-53-0	0.22 ± 0.06 ^b^	/	0.42 ± 0.04 ^a^	0.42 ± 0.01 ^a^
25	Acetic acid	64-19-7	4.54 ± 0.05 ^b^	6.22 ± 0.02 ^a^	3.56 ± 0.03 ^c^	3.47 ± 0.01 ^c^
	∑Acids		4.76 ± 0.11 ^b^	6.22 ± 0.02 ^a^	3.98 ± 0.07 ^b^	3.89 ± 0.02 ^b^
26	2,4-di-tert-Butylphenol	96-76-4	/	/	/	0.33 ± 0.03
27	2,6,10,10-tetramethyl-1-oxaspiro[4.5]dec-6-ene	36431-72-8	0.77 ± 0.02 ^c^	0.85 ± 0.02 ^b^	0.99 ± 0.03 ^a^	0.65 ± 0.04 ^d^
28	Cyclododecane	294-62-2	/	/	0.76 ± 0.27	/
29	1-decene	872-05-9	/	1.21 ± 0.31	/	/
	∑Other compounds		0.77 ± 0.02 ^d^	2.06 ± 31.02 ^b^	1.75 ± 0.30 ^a^	0.98 ± 0.07 ^c^

Note: “/” represents a compound that was not detected; values in the same column with different lowercase letters are significantly different (*p* < 0.05).

## Data Availability

The original contributions presented in this study are included in the article/Supplementary Material. Further inquiries can be directed to the corresponding author.

## Supplementary Materials

The following supporting information can be downloaded at: https://www.mdpi.com/article/10.3390/foods14040553/s1, Table S1. Diversity index of fungus in spontaneous fermentation of *Docynia delavayi* (Franch.) Schneid. Wine. Figure S1. H_2_S (A) and ethanol (B) production capacity of the selected yeasts isolated from *Docynia delavayi* (Franch.) Schneid.
