# Supplementary material for: Biodiversity and Winemaking Characteristics of Yeasts Isolated from Docynia delavayi (Franch.) Schneid. Wine Microbiota
Source: Foods. 2025 Feb 7;14(4):553. doi: 10.3390/foods14040553 (PMC11854437; doi:10.3390/foods14040553)
Supplement: Supplementary file 1 [file foods-14-00553-s001.zip › foods-3440327-supplementary.pdf]

Supplementary Materials for

Biodiversity and Winemaking Characteristics of Yeasts Isolated from *Docynia delavayi* (Franch.) Schneid. Wine Microbiota

Ling Zhu [1], Zhangxing Li [2], Yupeng Liang [3], Xiu Gao [1], Qingfang Xu [1], Weiliang Liu [1], Lifang Zhang [1] and Jian Cai [1,*]

[1] Yunnan Engineering Research Center of Fruit Wine, Qujing Normal University, Qujing 655011, China; lingzhu2021@mail.qjnu.edu.cn (L.Z.); appleartgao@163.com (X.G.); 101022@mail.qjnu.edu.cn (Q.X.); liuweiliang@mail.qjnu.edu.cn (W.L.); lifangz6933@mail.qjnu.edu.cn (L.Z.)
[2] Faculty of Food Science and Engineering, Kunming University of Science and Technology, Kunming 650500, China; 18206877260@163.com
[3] National Key Laboratory for Conservation and Utilization of Bio-Resources in Yunnan, Key Laboratory of Microbial Diversity in Southwest China, Ministry of Education, Yunnan Institute of Microbiology, School of Life Sciences, Yunnan University, Kunming 650091, China; lyp2019@mail.ynu.edu.cn
* Correspondence: caijian928@mail.qjnu.edu.cn; Tel./Fax: +86-874-899-8627

Table S1. Diversity index of fungus in spontaneous fermentation of *Docynia delavayi*（*Franch.*）Schneid. Wine.

Samples	Coverage	Shannon index	Simpson index	Sobs index	Ace index	Chao index
DY1	1.00	0.29	0.92	25.00	25.23	25.00
DY3	1.00	0.43	0.82	29.00	51.45	47.33
DY5	1.00	0.67	0.70	21.00	27.75	23.50
DY15	1.00	1.38	0.29	22.00	30.23	26.20

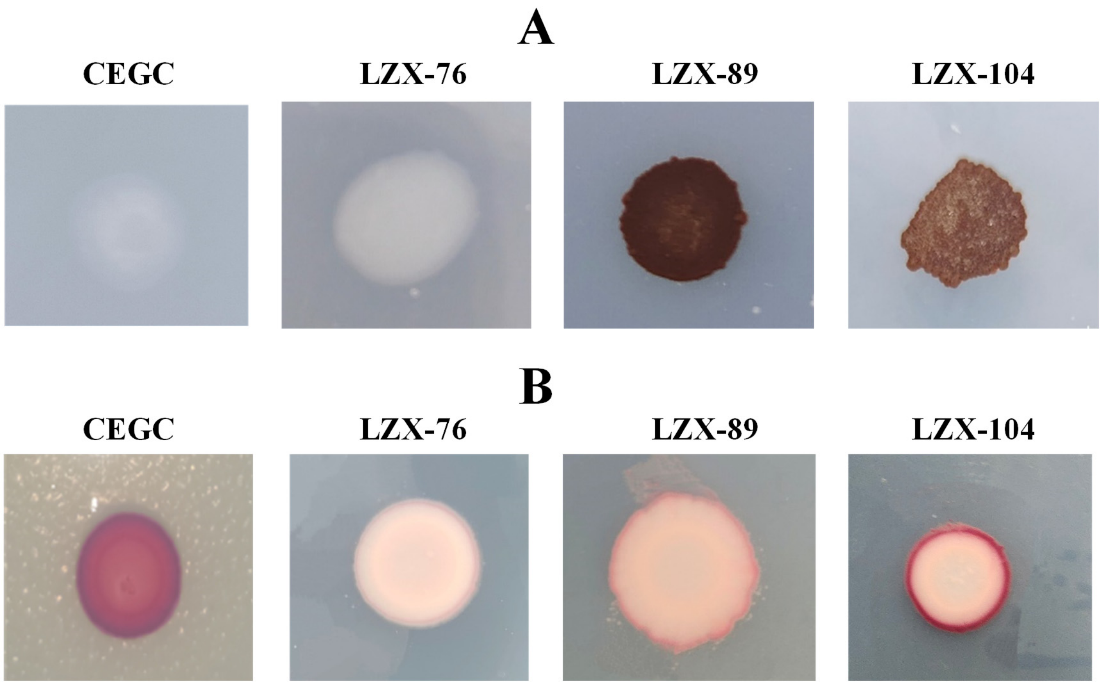

Figure S1 H_2S (A) and ethanol (B) production capacity of the selected yeasts isolated from *Docynia delavayi* (Franch.) Schneid.
